# Dual CRISPR interference and activation for targeted reactivation of X-linked endogenous *FOXP3* in human breast cancer cells

**DOI:** 10.1186/s12943-021-01472-x

**Published:** 2022-02-07

**Authors:** Xuelian Cui, Chao Zhang, Zhifang Xu, Shuaibin Wang, Xin Li, Erica Stringer-Reasor, Sejong Bae, Leiping Zeng, Dehua Zhao, Runhua Liu, Lei S. Qi, Lizhong Wang

**Affiliations:** 1 Department of Genetics, University of Alabama at Birmingham, 720 20th Street South, Birmingham, AL 35294 USA; 2 Department of O’Neal Comprehensive Cancer Center, University of Alabama at Birmingham, 720 20th Street South, Birmingham, AL 35294 USA; 3 Department of Medicine, University of Alabama at Birmingham, Birmingham, AL USA; 4 Department of Bioengineering, Stanford University, 443 Via Ortega, Stanford, CA 94305 USA; 5 Department of Chemical and Systems Biology, Stanford University, 443 Via Ortega, Stanford, CA 94305 USA; 6 ChEM-H Institute, Stanford University, 443 Via Ortega, Stanford, CA 94305 USA

**Keywords:** FOXP3, Breast cancer, CRISPR, X-linked gene, Transcript

## Abstract

**Background:**

Unlike autosomal tumor suppressors, X-linked tumor suppressors can be inactivated by a single hit due to X-chromosome inactivation (XCI). Here, we argue that targeted reactivation of the non-mutated allele from XCI offers a potential therapy for female breast cancers.

**Methods:**

Towards this goal, we developed a dual CRISPR interference and activation (CRISPRi/a) approach for simultaneously silencing and reactivating multiple X-linked genes using two orthogonal, nuclease-deficient CRISPR/Cas9 (dCas9) proteins.

**Results:**

Using *Streptococcus pyogenes* dCas9-KRAB for silencing *XIST* and *Staphylococcus aureus* dCas9-VPR for activating *FOXP3*, we achieved CRISPR activation of *FOXP3* in various cell lines of human female breast cancers. In human breast cancer HCC202 cells, which express a synonymous heterozygous mutation in the coding region of *FOXP3*, simultaneous silencing of *XIST* from XCI led to enhanced and prolonged *FOXP3* activation. Also, reactivation of endogenous *FOXP3* in breast cancer cells by CRISPRi/a inhibited tumor growth *in vitro* and *in vivo*. We further optimized CRISPRa by fusing dCas9 to the demethylase TET1 and observed enhanced *FOXP3* activation. Analysis of the conserved CpG-rich region of *FOXP3* intron 1 confirmed that CRISPRi/a-mediated simultaneous *FOXP3* activation and *XIST* silencing were accompanied by elevated H4 acetylation, including H4K5ac, H4K8ac, and H4K16ac, and H3K4me3 and lower DNA methylation. This indicates that CRISPRi/a targeting to *XIST* and *FOXP3* loci alters their transcription and their nearby epigenetic modifications.

**Conclusions:**

The simultaneous activation and repression of the X-linked, endogenous *FOXP3* and *XIST* from XCI offers a useful research tool and a potential therapeutic for female breast cancers.

**Supplementary Information:**

The online version contains supplementary material available at 10.1186/s12943-021-01472-x.

## Main text

Autosomal tumor suppressor genes can be inactivated by a two-hit Knudson mechanism. However, X chromosome-linked tumor suppressor genes, such as *FOXP3* at Xp11.23 [1], can be inactivated by a single-hit mechanism, because of X-chromosome inactivation (XCI). In female breast cancer cells, all identified gene deletions of *FOXP3* are heterozygous, and mice with a *Foxp3*-heterozygous mutation develop spontaneous breast cancers, suggesting that the active allele may be the only allele affected [1]. Thus, for females with cancer, it may be possible to reactivate the non-mutated, inactivated allele for therapeutic purposes. We recently developed, for complex gene regulation, a flexible endonuclease-deficient CRISPR/Cas9 (dCas9)-based platform that independently controls the expression of various genes (repression and activation) within the same cell [2]. Thus, for therapeutic purposes, targeted reactivation of XCI-endogenous tumor suppressor genes may be an effective strategy to restore their function in female cancer cells. The X-linked FOXP3 gene has dual roles in tumor cells and immune cells. As a master transcriptional regulator of regulatory T cells, FOXP3 limits antitumor immunity [3], whereas, in breast cancer cells, it is an epithelial cell-intrinsic tumor suppressor and is implicated in a tumor-suppressive function in the inhibition of tumor initiation and progression [1, 4–6]. Thus, using *FOXP3* as an X-linked model gene, we aimed to develop, for human female breast cancer cells, a tunable and reversible, targeted reactivation of X-linked tumor suppressor genes. In the present study, using CRISPR interference and activation (CRISPRi/a), we achieved, for human female breast cancer cells, targeted reactivation of X-linked endogenous *FOXP3*, at least a partial reactivation from XCI. Next, we investigated the potential epigenetic mechanism during CRISPRi/a-mediated reactivation of *FOXP3*.

## Results and discussion

### Transcription regulation of *XIST and FOXP3* by CRISPRi/a in human breast cancer cells

**Fig. 1:**
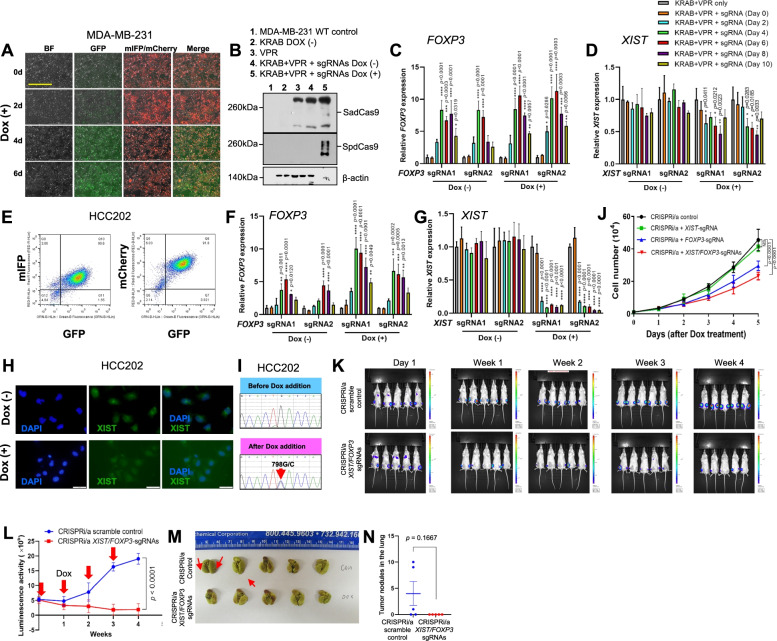
Assessment of CRISPRi/a in activation of endogenous *FOXP3* and repression of *XIST* in human breast cancer cells. **A** efficacy of co-transduction of the *XIST* (mIFP) and *FOXP3* (mCherry) sgRNAs in CRISPRi/a MDA-MB-231 cells before and after Dox addition at days 0, 2, 4, and 6 as determined by fluorescence microscopy. The expression of SpdCas9-KRAB (GFP) is induced by Dox in CRISPRi/a cells. BF, bright field. Scale bar, 1,000 μm. **B** protein expression of SpdCas9 and SadCas9 in CRISPRi/a MDA-MB-231 cells before and after sgRNA transduction and Dox treatment at days 0 and 4 as determined by Western blots with specific anti-SadCas9 and anti-SpCas9 antibodies. **C, D** quantitative expression analysis of *FOXP3* and *XIST* before and after sgRNA transduction and Dox treatment of CRISPRi/a MDA-MB-231 cells at days 0, 2, 4, 6, 8, and 10 as determined by qPCR. The fold change in mRNA expression was calculated using the 2^-ΔΔ Ct^ method with *GAPDH* mRNA as an internal control. **E** targeted cell sorting of the CRISPRi/a HCC202 cells after transduction of *XIST* (mIFP) and *FOXP3* (mCherry) sgRNAs and addition of Dox at day 4 as determined by flow cytometry. **F, G** quantitative expression analysis of *FOXP3* and *XIST* before and after sgRNA transduction and Dox addition in CRISPRi/a HCC202 cells at days 0, 2, 4, 6, 8, and 10 as determined by qPCR. **H** expression of *XIST* in CRISPRi/a cells after Dox addition for 4 days as determined by RNA fluorescence *in situ* hybridization analysis. Cells were hybridized to the *XIST* probe (green). DAPI (blue) was used as a nuclear counterstain. DAPI, 4’,6-diamidino-2-phenylindole. Scale bar, 50 μm. **I** mutation analysis of a 798G/C mutation (red arrow) of the human *FOXP3* transcript in CRISPRi/a HCC202 cells before and after activation of *FOXP3* as determined by cDNA sequencing. **J** effect of CRISPRi/a-induced endogenous *FOXP3* on growth of HCC202 cells. Cell proliferation was measured after Dox treatment. **K** bioluminescent and optical images of CRISPRi/a (with *FOXP3/XIST* sgRNAs) and scrambled (CRISPRi/a without *FOXP3/XIST* sgRNAs) HCC202 cells using the In Vivo Bio-luminescence Imaging System. Eight-week-old NSG female mice were performed by intratibial injection with 1x10^5^ CRISPRi/a and scrambled HCC202 luciferase cells, respectively, followed by Dox injection (2.5 mg/kg weekly). **L** Quantitative analysis of the *ex vivo* bioluminescent imaging results from panels in **K**. **M** Representative optical images of lung metastasis in mice with CRISPRi/a and scrambled control cells at day 28 after bone transplantation. Red down arrows indicate Dox injection. **N** Quantification of lung metastatic nodules. The number of surface tumor lesions over all lobes of the lungs was scored for metastatic nodules. Data are presented as the means ± standard deviation. *p* values were determined by ANOVA followed by Tukey's *post hoc* test, Mann Whitney test or by two-way ANOVA test *vs*. control group. All experiments were repeated three times

Since most human female breast cancer cell lines have low or no *FOXP3* expression [1], accompanied by heterozygous gene deletions but rare *FOXP3* mutations (Table S1), it may be possible to reactivate *FOXP3* from XCI. We determined the expression levels of *X-inactive specific transcript* (*XIST*) in various female breast cancer cell lines, including MDA-MB-231, MCF7, and HCC202. As shown in Fig. S1, expression of *XIST* was high in HCC202 cells, but low in MCF7 and MDA-MB-231 cells. To achieve simultaneous transcriptional repression of *XIST* and reactivation of *FOXP3* in the same cells, we utilized two orthogonal CRISPR/dCas9 systems [2], *Streptococcus pyogenes* (Sp) dCas9-Krüppel-associated box (KRAB) (SpdCas9-KRAB) for silencing *XIST* and *Staphylococcus aureus* (Sa) dCas9-tripartite VP64-p65-Rta proteins (VPR) (SadCas9-VPR) for reactivation of *FOXP3* (Figs. S2A and B). We established the CRISPRi/a MDA-MB-231 cell model stably expressing SadCas9-VPR and SpdCas9-KRAB (Table S2). Then, we transiently co-transduced *XIST*- and *FOXP3*-single guide RNAs (sgRNAs) into the CRISPRi/a MDA-MB-231 cells (Figs. S2C-E). The efficacy of transduction of sgRNAs in the cells was validated by fluorescence microscopy (Fig. 1A) and Western blots (Fig. 1B). After transduction of sgRNAs, quantitative real-time PCR (qPCR) analysis showed that levels of the *FOXP3* transcript were increased 8-fold by *FOXP3*-sgRNA with *XIST*-sgRNA at day 4 and up to 8 days (Fig. 1C). However, after doxycycline (Dox) addition, levels of the *XIST* transcript were reduced by *XIST*-sgRNA, but this difference was not statistically significant (Fig. 1D). Likewise, levels of the *FOXP3* transcript in MDA-MB-231 cells were not significantly different after addition of Dox (Fig. 1C), suggesting a dominant activation of the *FOXP3* transcript from an active X-linked allele. Furthermore, CRISPRi/a (with *FOXP3/XIST*-sgRNAs) and scrambled (CRISPRi/a without *FOXP3/XIST*-sgRNAs) MDA-MB-231 cells were injected into the fourth mammary fat pads of 8-week-old NSG female mice followed by Dox injection weekly for 28 days. As shown in Fig. S2F-H, xenograft tumor growth of CRISPRi/a MDA-MB-231 cells was slower than that of scrambled MDA-MB-231 cells. Also, reactivation of *FOXP3* in xenograft tumors by CRISPRi/a was validated by qPCR (Fig. S2I). Next, we established the CRISPRi/a MCF7 cell model and transiently co-transduced both *XIST*- and *FOXP3*-sgRNAs into these cells (Fig. S3A and B and Table S2). After transduction of sgRNAs, levels of the *FOXP3* transcript were increased more than 8-fold by the *FOXP3*-sgRNA with *XIST*-sgRNA (Fig. S3C). However, levels of the *FOXP3* transcript in the cells were not changed by Dox, although levels of the *XIST* transcript were reduced by Dox (Fig. S3D), suggesting activation of the *FOXP3* transcript from an active X-linked allele.

### CRISPRi/a-mediated targeted reactivation of X-linked *FOXP3* from XCI in human breast cancer cells

The HCC202 cell line expresses a synonymous heterozygous mutation (p.L266L

c.798G>C) in the coding region of *FOXP3* (Table S1), enabling us to determine, by cDNA sequencing, if *FOXP3* is reactivated from one or both alleles. Also, in HCC202 cells, the two alleles of *FOXP3* showed no deletion, but there were low expression levels of *FOXP3* (Table S1). Thus, we established the CRISPRi/a HCC202 cell model (Fig. S4A and Table S2). After being transiently co-transduced with both *XIST*- and *FOXP3*-sgRNAs, the sgRNA-transduced cells were sorted by flow cytometry (Fig. 1E). After sgRNA transductions, levels of the *FOXP3* transcript were increased approximately 6-fold by the *FOXP3*-sgRNA with *XIST*-sgRNA (Fig. 1F). After addition of Dox, transduction of *XIST* sgRNA reduced more than 90% of *XIST* expression at day 2 and up to day 10 (Fig. 1G and H). Simultaneously, levels of the *FOXP3* transcript were elevated approximately 2-fold at day 4 after Dox addition (Fig. 1F). Of note, by cDNA sequencing, a heterozygous 798G/C mutation of *FOXP3* was identified in Dox-treated CRISPRi/a-*FOXP3*/*XIST* HCC202 cells but not in untreated CRISPRi/a-*FOXP3*/*XIST* HCC202 cells (Fig. 1I), suggesting that, in HCC202 cells, CRISPRi/a-induced activation of the *FOXP3* transcript is at least partially reactivated from XCI under *XIST* downregulation. In addition, to test the effect of CRISPRi/a-induced *FOXP3* reactivation on FOXP3 target genes in HCC202 cells, we assessed the expression of *p21* (*CDKN1A*) and *SKP2,* which are prominent transcriptional targets of FOXP3 in breast cancer cells [7, 8]. Our data revealed that, for HCC202 cells, reactivation of endogenous *FOXP3* by CRISPRi/a induced the transcription of *p21* but reduced the transcription of *SKP2* (Fig. S4B and C). Of note, Dox-induced reactivation of *FOXP3* from XCI enhanced the transcriptional regulation of *p21* and *SKP2* in HCC202 cells.

Using the established CRISPRi/a HCC202 cells, we determined the effect of CRISPRi/a-induced endogenous *FOXP3* on cell growth. We transiently transduced *XIST* sgRNA, *FOXP3* sgRNA, or both into CRISPRi/a HCC202 cells for 48 hours, and then added Dox to the cells for 5 days. As shown in Figs. S4D and E, levels of the *FOXP3* transcript were gradually elevated in the CRISPRi/a cells with *FOXP3* sgRNA, whereas levels of *XIST* were reduced in the CRISPRi/a cells with *XIST* sgRNA from days 1 to 5 after addition of Dox. Cell growth was slower for CRISPRa cells with *FOXP3* sgRNA and slowest for CRISPRi/a cells with *FOXP3/XIST* sgRNAs relative to CRISPRi/a control cells, but there was no difference between cells with *XIST* sgRNA alone and control CRISPRi/a HCC202 cells (Fig. 1J), suggesting that CRISPRi/a-induced endogenous *FOXP3* inhibits growth of HCC202 cells. Furthermore, we injected CRISPRi/a HCC202 cells with or without *FOXP3/XIST*-sgRNAs into the fourth mammary fat pads of 8-week-old NSG female mice. However, we failed to generate orthotopic xenograft tumors using CRISPRi/a and scrambled HCC202 cells until day 28 after injection. Since bone is a common metastatic site for patients with breast cancer [9], we injected the luciferase-transduced CRISPRi/a and scrambled HCC202 cells into the tibia bones of 8-week-old NSG female mice followed by weekly Dox injections. As shown in Fig. 1K and L, luciferase imaging analysis showed that xenograft tumor growth in the bone was slower for CRISPRi/a cells (CRISPRi/a with *FOXP3/XIST*-sgRNAs) compared to scramble cells (CRISPRi/a without *FOXP3/XIST*-sgRNAs) for 28 days after tumor cell injection, supporting tumor growth inhibition by CRISPRi/a-induced endogenous *FOXP3 in vivo*. Likewise, lung metastases were evident in 60% of the mice (3/5) with CRISPRi/a cells but were not observed in mice (0/5) injected with scramble cells (Fig. 1M). However, due to a small sample size, quantitative analysis of tumor nodules in the lung showed no significant difference between mice with CRISPRi/a cells and scramble cells (*p* = 0.1667, Fig. 1N).

To exclude off-target effects of our CRISPRi/a, we evaluated the potential off-target genes of our designed sgRNAs using the Cas-OFFinder (Table S3a-d). For *FOXP3*-sgRNA, the three nucleotide mismatched genes, *CFAP61*, *ERI3*, and *ZFAT*, were assessed by qPCR. Although expression levels of the potential off-target genes undulated in the CRISPRi/a cells with *FOXP3* sgRNAs, overall changes in these genes were not significant from days 0 to 10 after Dox addition (Fig. S5A-C). Likewise, for *XIST*-sgRNA, the three nucleotide mismatched genes, *ASXL2*, *IGF2BP2*, and *VAMP4*, were not significantly changed from days 0 to 10 after Dox addition (Fig. S5D-F).

### Effect of DNA demethylation on CRISPRi/a-mediated activation of *FOXP3* in human breast cancer cells

**Fig. 2:**
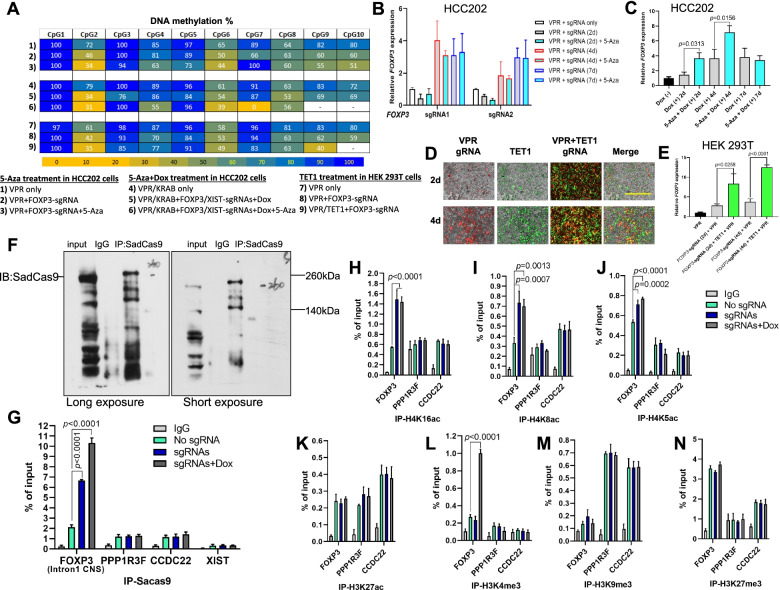
DNA methylation status and histone modifications in the conserved CNS of *FOXP3* intron 1 during reactivation of *FOXP3* in human CRISPRi/a cells. **A** heatmap of DNA methylation status of rich CpG sites in the conserved CNS of *FOXP3* intron 1 during reactivation of *FOXP3* in human HCC202 CRISPRi/a or HEK 293T CRISPRa cells determined by pyrosequencing. **B** quantitative expression analysis of *FOXP3* before and after sgRNA transduction and 5-Aza-2'-deoxycytidine (5-Aza) treatment of CRISPRa HCC202 cells at days 0, 2, 4, and 7 as determined by qPCR. **C** quantitative expression analysis of *FOXP3* before and after sgRNAs transduction and 5-Aza and Dox treatment of CRISPRi/a HCC202 cells at days 0, 2, 4, and 7 as determined by qPCR. **D** efficacy of transduction of *FOXP3* sgRNA (mCherry) and transfection of SadCas9-TET1 (GFP) into CRISPRa HEK 293T cells at days 2 and 4 as determined by fluorescence microscopy. Scale bar, 1,000 μm. **E** quantitative expression analysis of *FOXP3* before and after sgRNA transduction and SadCas9-TET1 transfection into CRISPRa HEK 293T cells at days 0, 2, and 4 as determined by qPCR. The fold change in expression was calculated using the 2^-ΔΔ Ct^ method with *GAPDH* mRNA as an internal control. **F** IP with a specific anti-SadCas9 antibody (left panel: long exposure; right panel: short exposure) in CRISPRi/a HEK 293T cells. **G** SadCas9 ChIP-qPCR in the conserved CNS of *FOXP3* intron 1 during CRISPRi/a-mediated activation of *FOXP3* with or without *XIST-* and *FOXP3*-sgRNAs and Dox at day 4 in HEK 293T cells. The Y-axis represents % of input DNA. The values for the binding of SadCas9 to targeted locus were normalized to total input genomic DNA of each region. **H-N** various histone ChIP-qPCR analyses in the conserved CNS of *FOXP3* intron 1 during CRISPRi/a-mediated activation of *FOXP3* with or without *XIST-* and *FOXP3*-sgRNAs and Dox at day 4 in HEK 293T cells. Relative histone enrichment levels were normalized to total input genomic DNA of each region. The promoter loci of *FOXP3* neighboring genes, *PPP1R3F* and *CCDC22*, were used as reference controls. Data are presented as the means ± standard deviation. *p* values were determined by a two-tailed *t*-test between two groups or ANOVA followed by Tukey’s *post hoc* test *vs.* a control group. All experiments were repeated three times

*XIST* RNA works in concert with DNA methylation and histone modifications to maintain XCI [10, 11]. In regulatory T cells, DNA demethylation of the conserved non-coding sequence (CNS) of the *FOXP3* intron 1 is specific for inducing or stabilizing transcription of *FOXP3* [12–15]. First, we determined whether treatment with the DNA methylation inhibitor, 5-aza-2'-deoxycytidine (5-Aza-CdR), enhanced CRISPRi/a-mediated activation of *FOXP3* in human breast cancer cells. As shown in Fig. S6A and B, we transiently transduced *FOXP3*-sgRNAs into CRISPRa HCC202 cells, followed by treatment with or without 5-Aza-CdR. For the 10 CpG sites of conserved CNS of *FOXP3* intron 1, pyrosequencing analysis showed deregulation of DNA methylation by CRISPRa, 5-Aza-CdR, or both, in 7/10 CpG sites, but these changes appeared to be not statistically significant (Figs. 2A and S7A-C). Likewise, *FOXP3* was induced in the cells after *FOXP3*-sgRNA transduction, but levels of the *FOXP3* transcript were not changed by treatment with 5-Aza-CdR (Fig. 2B). Next, we transiently co-transduced *FOXP3/XIST*-sgRNAs into CRISPRi/a HCC202 cells, followed by treatment with or without 5-Aza-CdR and Dox addition (Fig. S6C and D). Treatment of *FOXP3*/*XIST*-sgRNAs-transduced CRISPRi/a cells with 5-Aza-CdR enhanced levels of the *FOXP3* transcript approximately 2-fold at days 2 and 4 (Fig. 2C), suggesting that induction of *FOXP3* by 5-Aza-CdR from XCI was under *XIST* downregulation. Likewise, pyrosequencing analysis revealed deregulation of DNA methylation by CRISPRi/a, 5-Aza-CdR, or both in most CpG sites (Figs. 2A and S7D).

The ten-eleven translocation (TET) family of DNA demethylase proteins converts cytosine methylated at C5 (5mC) to 5hmC, 5fC, and 5caC, and finally to cytosine with the aid of thymine-DNA glycosylase [16]. These changes are associated with elevated gene transcription [17]. Thus, we constructed SadCas9-TET1 (Fig. S8A and B) for targeted DNA demethylation in the *FOXP3* CNS locus to enhance the activation of *FOXP3* in human breast cancer cells. However, we failed to transfect SadCas9-TET1 into the CRISPRa HCC202 cells due to the large construct size. Next, we transfected the SadCas9-TET1 and transduced the *FOXP3*-sgRNA into CRISPRa HEK 293T cells (Fig. S6E). For these cells, the efficacies of transfection and transduction were validated by fluorescence microscopy (Fig. 2D). After 2 days of transfection, Western blots confirmed protein expression of SadCas9-TET1 in the transfected cells (Fig. S8C). On days 2 and 4, levels of the *FOXP3* transcript were elevated approximately 3-fold in SadCas9-TET1-transfected and *FOXP3*-sgRNA transduced cells relative to cells transduced with *FOXP3*-sgRNA (Fig. 2E), suggesting, for HEK 293T cells, synergistically enhanced activation of *FOXP3* by co-expression of TET1 and VPR. Likewise, pyrosequencing analyses validated, for most CpG sites, deregulation of DNA methylation by CRISPRa with VPR, TET1, or both (Figs. 2A and S7E).

### Histone modifications during CRISPRi/a-mediated activation of *FOXP3* in human breast cancer cells

Histone methylation and acetylation either repress or activate transcription [18]. First, using established the CRISPRi/a HEK 293T cell models (Fig. S9A-D and Table S2), we performed chromatin immunoprecipitation (ChIP)-qPCR assays with a SadCas9-specific antibody (Fig. 2F). As shown in Fig. 2G, binding of SadCas9-VPR to the intron 1 CNS locus of *FOXP3* was elevated more than 3-fold in cells transduced with *FOXP3/XIST*-sgRNAs relative to cells without sgRNAs; this binding was enhanced after addition of Dox to the cells. Although SadCas9-VPR also bound to *FOXP3* neighbor genes, *PPP1R3F* and *CCDC22*, these bindings were not elevated after transduction of *FOXP3/XIST*-sgRNAs or addition of Dox to the cells (Fig. 2G). Further, expressions of *PPP1R3F* and *CCDC22* in the cells were not changed after the transduction of *FOXP3/XIST*-sgRNAs and addition of Dox (Fig. S10A and B). In addition, no specific binding of SadCas9-VPR was evident in the *XIST* locus (Fig. 2G). These data suggest a *FOXP3*-sgRNA-guided specific binding of SadCas9-VPR to the intron 1 CNS locus of *FOXP3.*

To address the histone modification in the intron 1 CNS locus of *FOXP3* during activation of *FOXP3*, we performed a ChIP-qPCR assay using CRISPRa-*FOXP3/XIST*-sgRNAs HEK 293T cells. As shown in Fig. 2H-N, in CRISPRi/a cells after *FOXP3/XIST*-sgRNAs transduction and Dox addition, H4K5ac, H4K8ac, and H4K16ac were enriched in the intron 1 CNS locus of *FOXP3* but not in the promoter regions of *PPP1R3F* and *CCDC22.* Of note, during activation of *FOXP3*, H4K8ac and H4K16ac were elevated more than 2-fold in the intron 1 CNS locus (Fig. 2H and I), but there were no significant changes after Dox addition. H3K4me3, H3K9me3, H3K27me3, and H3K27ac were minimally changed in the *FOXP3*, *PPP1R3F*, and *CCDC22* loci after *FOXP3/XIST*-sgRNAs transduction (Fig. 2K-N). However, H3K4me3 was elevated more than 3-fold in the intron 1 CNS locus after addition of Dox (Fig. 2L). These data suggest that, during activation of *FOXP3*, *FOXP3*-sgRNA guided specific H4 acetylation at active alleles and H3K4 methylation at inactive alleles in the intron 1 CNS locus of *FOXP3*.

As reported here, we developed, for human female breast cancer cells, a CRISPRi/a approach for targeted transcriptional regulation of specific X-linked *FOXP3*, using two orthogonal dCas9-fusion systems, including SpdCas9-KRAB for CRISPRi to the *XIST* locus and SadCas9-VPR for CRISPRa to the *FOXP3* locus (Fig. S11). The targeted reactivation of endogenous *FOXP3* from XCI was achieved by simultaneous use of CRISPRi/a. Of note, targeted reactivation of *FOXP3* inhibited growth of human female breast cancer cells. Furthermore, we optimized our CRISPRa system with the TET1 catalytic domain to enhance the transcriptional activation of *FOXP3*. The CRISPRi/a-mediated activation of *FOXP3* was accompanied by H4 acetylation at active alleles, including H4K5ac, H4K8ac, and H4K16ac, and H3K4 methylation at inactive alleles in the intron 1 CNS locus of *FOXP3*, indicating a CRISPRi/a-mediated epigenetic mechanism during activation of *FOXP3*.

## Conclusions

The present study provides a better understanding of the CRISPRi/a-mediated activation of X-linked endogenous *FOXP3* and its regulatory mechanism in human female breast cancer cells. Also, our identification of the reactivation of the X-linked *FOXP3* from XCI moves beyond an incremental advance in breast cancer therapy by a targeted reactivation of X-linked tumor suppressor genes. Since epithelial *FOXP3* is inactivated in 70% of breast cancer samples [1], our results may lead to the design of preclinical studies to develop more effective treatments for female breast cancers with *FOXP3* dysfunction. In addition, this concept and tools may provide new routes of targeted therapy for other X-chromosome-linked genetic disorders.

## Supplementary Information

**Additional file 1: Figure S1**. Expression of *XIST* in human embryonic kidney (HEK) 239T cells and breast cancer cell lines. The expression levels of *XIST* were assessed by qPCR. The fold change in expression was calculated using the 2^-ΔΔ Ct^ method with *GAPDH* mRNA as an internal control. Data are presented as means ± standard division (SD). HEK 293T, a human embryonic kidney 293 cell line with the SV40 T-antigen; MCF7, a human estrogen receptor (ER)-positive breast cancer cell line; MDA-MB-231, a human triple-negative breast cancer (TNBC) cell line; HCC202, a human epidermal growth factor receptor 2 (HER2)-positive breast cancer cell line. All experiments were repeated three times. **Figure S2.** CRISPRi/a DNA construction, experimental procedure, and targeted reactivation of endogenous *FOXP3 in vivo* in activation of endogenous *FOXP3* and repression of *XIST* in human breast cancer MDA-MB-231 cells. **A, B** diagrams showing the constructs of CRISPRi/a, including *S. pyogenes* (Sp) dCas9-KRAB (SpdCas9-KRAB) and *S. aureus* (Sa) dCas9-VPR (SadCas9-VPR) used in the experiment. **C** sgRNAs 1/2/3 targeted to the -50 to +300 bp upstream of the transcription start site of the *XIST* locus for transcription repression. **D** sgRNAs 1/2/3/4/5 targeted to the two CpG sites of the *FOXP3* proximal promoter and the intron 1 regions for transcription activation. **E** CRISPRi/a experimental procedure for the co-transduction of *XIST* (mIFP)- and *FOXP3* (mCherry)-sgRNAs, Dox induction, and targeted cell sorting of SpdCas9-KRAB (GFP after Dox) and SadCas9-VPR stably expressing MDA-MB-231 cells. CRISPRi, CRISPR interference; CRISPRa, CRISPR activation; sgRNA, single guide RNA; Dox, doxycycline; KRAB, transcription repressor Krüppel associated box for CRISPRi; VPR, transcription activators VP64-p65-Rta for CRISPRa. **F** CRISPRi/a MDA-MB-231 xenograft tumor growth in NSG mice (n=9/group). Solid black arrows indicate Dox injections. **G** xenograft tumors and weights at day 28. **I** the expression levels of *FOXP3* in xenograft tumors were assessed by qPCR. The fold change in expression was calculated using the 2^-ΔΔ Ct^ method with *GAPDH* mRNA as an internal control. Data are presented as means ± SD. *p* values by a two-way ANOVA or a two-tailed *t*-test. **Figure S3.** Assessment of CRISPRi/a to activate endogenous *FOXP3* and repress *XIST* in human breast cancer MCF7 cells. **A** CRISPRi/a experimental procedure for the co-transduction of *XIST* (mIFP)- and *FOXP3* (mCherry)-sgRNAs and expression of SpdCas9-KRAB (GFP) by Dox induction in CRISPRi/a MCF7 cells. **B** efficacy of co-transduction of the *XIST-* and *FOXP3-*sgRNAs in CRISPRi/a cells before and after Dox induction at days 4 and 8 as determined by fluorescence microscopy. Scale bar, 1,000 μm. **C, D** quantitative expression analysis of *FOXP3* and *XIST* before and after sgRNA transduction and Dox induction of CRISPRi/a cells at days 0, 2, 4, 6, 8, and 10 as determined by qPCR. The fold change in expression was calculated using the 2^-ΔΔ Ct^ method with *GAPDH* mRNA as an internal control. Data are presented as the means ± SD. *p* values by ANOVA followed by Tukey's *post hoc* test *vs.* the KRAB+VPR-only group. All experiments were repeated three times. **Figure S4.** CRISPRi/a DNA construction and experimental procedure and assessment of CRISPRi/a to repress *XIST* in activation of endogenous *FOXP3* and repression of *XIST* in human breast cancer HCC202 cells. **A** CRISPRi/a experimental procedure for the co-transduction of *XIST* (mIFP)- and *FOXP3* (mCherry)-sgRNAs and Dox induction (GFP for SpdCas9-KRAB) in CRISPRi/a HCC202 cells. **B, C** quantitative expression analysis of *p21* and *SKP2* by qPCR in CRISPRi/a HCC202 cells with or without Dox at days 0, 2, 4, 6, 8, and 10. **D, E** quantitative expression analysis of *FOXP3* and *XIST* by qPCR in CRISPRi/a HCC202 cells with or without Dox. The fold change in expression was calculated using the 2^-ΔΔ Ct^ method with *GAPDH* mRNA as an internal control. Error bars, SD. *p* values by a two-way ANOVA or one-way ANOVA followed by Tukey's analysis. All experiments were repeated three times. **Figure S5.** Assessment of potential off-target genes of *FOXP3* and *XIST* sgRNAs in CRISPRi/a HCC202 cells. Quantitative expression analysis of the potential off-target genes of *FOXP3* sgRNAs (**A-C**) and *XIST* sgRNAs (**D-F**) before and after sgRNA transduction and Dox induction in CRISPRi/a HCC202 cells at days 0, 2, 4, 6, 8, and 10 as determined by qPCR. The fold change in expression was calculated using the 2^-ΔΔ Ct^ method with *GAPDH* mRNA as an internal control. Data are presented as the means ± SD. * *p* < 0.05 by one-way ANOVA followed by Tukey’s *post hoc* test. All experiments were repeated three times. **Figure S6**. Experimental procedure and efficacy of transduction in various cell models. **A** the CRISPRi/a experimental procedure for the transduction of *FOXP3* sgRNA (mCherry) with or without 5-Aza-CdR (5-Aza) in CRISPRa HCC202 cells. **B,** efficacy of transduction of *FOXP3* sgRNA in CRISPRa HCC202 cells before and after 5-Aza treatment at days 2, 4, and 7 as determined by fluorescence microscopy. Scale bar, 100 μm. **C** CRISPRi/a experimental procedure for the co-transduction of *XIST* (mIFP)- and *FOXP3* (mCherry)-sgRNAs with or without 5-Aza and Dox for CRISPRa HCC202 cells. **D** efficacy of co-transduction of the *FOXP3* sgRNA in CRISPRa HCC202 cells before and after 5-Aza and Dox treatment at days 2, 4, and 7 as determined by fluorescence microscopy. Scale bar, 1,000 μm. **E** CRISPRa experimental procedure for the transduction of *FOXP3* sgRNA (mCherry) with or without SadCas9-TET1 (GFP) into CRISPRa HEK 293T cells. **Figure S7**. DNA methylation status of the rich CpG sites in conserved CNS of *FOXP3* intron 1 during CRISPRi/a-mediated activation of *FOXP3* in human female cells. **A** diagram of the 10 CpG sites in conserved CNS of *FOXP3* intron 1 and bisulfite PCR design for DNA methylation pyrosequencing analysis. **B** DNA methylation analysis by pyrosequencing for CRISPRa HCC202 cells, CRISPRi/a HCC202 cells, and CRISPRa HEK 293T cells with various treatments. Pyrosequencing was performed to measure the methylation levels at 10 CpG sites in the conserved CNS of *FOXP3* intron 1 using the PyroMark Q96 ID pyrosequencer. **C-E** average levels of DNA methylation in CRISPRa HCC202 cells, CRISPRi/a HCC202 cells, and CRISPRa HEK 293T cells before and after various treatments. Data are presented as the means ± SD. 5-Aza, 5-Aza-2’-deoxycytidine. All experiments were repeated three times. **Figure S8**. Establishment of SadCas9-TET1 DNA constructs. **A** schematic construction of the SadCas9-TET1 vector used in the experiment. **B** horizontal gel electrophoresis analysis of bands of the TET1 catalytic domain and VPR digested from SadCas9-TET1 and SadCas9-VPR vectors, respectively. Molecular sizes of the 10-kb DNA ladder are indicated on the left side. **C** Protein expression of TET1 after transfection into HEK 293T cells. The SadCas9-TET1 vector was transiently transfected into HEK 293T cells. The red arrow indicates the size of the SadCas9-TET1 catalytic domain. The blue arrow indicates the full size of the endogenous TET1 protein. IB, Immunoblotting. **Figure S9**. Targeted activation of FOXP3 and inactivation of XIST in human embryonic kidney (HEK) 293T cells. **A** CRISPRi experimental procedure for the transduction of *XIST* sgRNA (mIFP), Dox induction, and targeted cell sorting of SpdCas9-KRAB (GFP after Dox) stably expressing HEK 293T cells. **B** CRISPRa experimental procedure for the transduction of *FOXP3* sgRNA (mCherry), Dox induction, and targeted cell soring of SpdCas9-KRAB (GFP after Dox) stably expressing HEK 293T cells. **C, D** quantitative expression analysis of *XIST* and *FOXP3* by qPCR of CRISPRi and CRISPRa cells, respectively. After Dox (1.0 μg/ml) induction, the expression levels of *XIST* and *FOXP3* in the transduced cells were determined at days 0, 2, 4, 6, 8, and 10. The fold change in expression was calculated using the 2^-ΔΔ Ct^ method with *GAPDH* mRNA as an internal control. Data are presented as the means ± standard deviation (SD). *p* values by a one-way ANOVA test. CRISPRi, CRISPR interference; CRISPRa, CRISPR activation; sgRNA, single guide RNA; Dox, doxycycline; KRAB, transcription repressor Krüppel associated box for CRISPRi; VPR, transcription activators VP64-p65-Rta for CRISPRa. All experiments were repeated three times. **Figure S10.** Effect of CRISPRi/a-mediated activation of *FOXP3* on expression of its neighboring genes in HCC202 cells. *PPP1R3F* and *CCDC22* are two *FOXP3* neighboring genes at Xp11.23. Quantitative expression analysis of *CCDC22* (**A**) and *PPP1R3F* (**B**) before and after sgRNA transduction and Dox induction in the CRISPRi/a cells at days 0, 2, 4, 6, 8, and 10 as determined by qPCR. The fold change in expression was calculated using the 2^-ΔΔ Ct^ method with *GAPDH* mRNA as an internal control. Data are presented as the means ± SD. *p* values by one-way ANOVA followed by Tukey’s *post hoc* test. All experiments were repeated three times. **Figure S11.** Targeted reactivation of the X-linked endogenous FOXP3 gene from X chromosome inactivation (XCI) in female cells. PRC (polycomb repressive complex) 1 or 2 recruits *XIST* RNA and promotes epigenetic modifications that block X-linked FOXP3 gene transcription on the inactive X chromosome. DNA binding by SadCas9-VPR (VP64/p65/Rta) and SadCas9-TET to the *FOXP3* intron 1 enhancer, and subsequent epigenetic modifications, in conjunction with the SpdCas9-KRAB to the *XIST* promoter, reactivates X-linked FOXP3 gene transcription from XCI.

**Additional file 2.** Materials and Methods

**Additional file 3: Table S1.** Gene copy number, mutation and expression of the FOXP3 gene in human breast cancer cell lines

**Additional file 4: Table S2.** CRISPR/dCas9-KRAB-, VPR-, *XIST*-sgRNA, and *FOXP3*-sgRNA stably expressed cell models

**Additional file 5: Table S3a.** The potential off-targets of *FOXP3* accessed using the Cas-OFFinder off-target searching tool. **b.** The potential off-targets of *XIST* accessed using the Cas-OFFinder off-target searching tool. **c.** The potential mismatch ≤4 off-target regions accessed using the Cas-OFFinder off-target searching tool. **d.** The potential mismatch ≤4 off-target regions accessed using the Cas-OFFinder off-target searching tool.

**Additional file 6: Table S4.** CRISPR/dCas9 DNA constructs for targeted repression of *XIST* and activation of *FOXP3*

**Additional file 7: Table S5.** The sequences of PCR primers and CRISPR sgRNAs used in this study

**Additional file 8: Table S6.** Specific primary antibodies used in this study

## Data Availability

Results are based, in part, upon data generated by Cas-OFFinder (http://www.rgenome.net/cas-offinder).

## Abbreviations

5-Aza-CdR: 5-aza-2’-deoxycytidine
ChIP: Chromatin immunoprecipitation
CNS: Conserved non-coding sequence
CRISPRa: CRISPR activation
CRISPRi: CRISPR interference
dCas9: Endonuclease-deficient CRISPR/Cas9 protein
Dox: Doxycycline
H3K4me3: Tri-methylation at the 4th lysine residue of the histone H3 protein
H3K9me3: Tri-methylation at the 9th lysine residue of the histone H3 protein
H3K27ac: Acetylation at the 27th lysine residue of the histone H3 protein
H3K27me3: Tri-methylation at the 27th lysine residue of the histone H3 protein
H4K5ac: Acetylation at the 5th lysine residue of the histone H4 protein
H4K8ac: Acetylation at the 8th lysine residue of the histone H4 protein
H4K16ac: Acetylation at the 16th lysine residue of the histone H4 protein
KRAB: Krüppel-associated box
qPCR: Quantitative real-time PCR
Sa: *Staphylococcus aureus*
sgRNA: Single guide RNA
Sp: *Streptococcus pyogenes*
TET: Ten-eleven translocation
VPR: Tripartite VP64-p65-Rta proteins
XCI: X-chromosome inactivation
XIST: X-inactive specific transcript
