# Supplementary material for: Dual CRISPR interference and activation for targeted reactivation of X-linked endogenous FOXP3 in human breast cancer cells
Source: Mol Cancer. 2022 Feb 7;21:38. doi: 10.1186/s12943-021-01472-x (PMC8819949; doi:10.1186/s12943-021-01472-x)
Supplement: Supplementary file 1 — Additional file 1: Figure S1. Expression of XIST in human embryonic kidney (HEK) 239T cells and breast cancer cell lines. The expression levels of XIST were assessed by qPCR. The fold change in expression was calculated using the 2-ΔΔ Ct method with GAPDH mRNA as an internal control. Data are presented as means ± standard division (SD). HEK 293T, a human embryonic kidney 293 cell line with the SV40 T-antigen; MCF7, a human estrogen receptor (ER)-positive breast cancer cell line; MDA-MB-231, a human triple-negative breast cancer (TNBC) cell line; HCC202, a human epidermal growth factor receptor 2 (HER2)-positive breast cancer cell line. All experiments were repeated three times. Figure S2. CRISPRi/a DNA construction, experimental procedure, and targeted reactivation of endogenous FOXP3 in vivo in activation of endogenous FOXP3 and repression of XIST in human breast cancer MDA-MB-231 cells. A, B diagrams showing the constructs of CRISPRi/a, including S. pyogenes (Sp) dCas9-KRAB (SpdCas9-KRAB) and S. aureus (Sa) dCas9-VPR (SadCas9-VPR) used in the experiment. C sgRNAs 1/2/3 targeted to the -50 to +300 bp upstream of the transcription start site of the XIST locus for transcription repression. D sgRNAs 1/2/3/4/5 targeted to the two CpG sites of the FOXP3 proximal promoter and the intron 1 regions for transcription activation. E CRISPRi/a experimental procedure for the co-transduction of XIST (mIFP)- and FOXP3 (mCherry)-sgRNAs, Dox induction, and targeted cell sorting of SpdCas9-KRAB (GFP after Dox) and SadCas9-VPR stably expressing MDA-MB-231 cells. CRISPRi, CRISPR interference; CRISPRa, CRISPR activation; sgRNA, single guide RNA; Dox, doxycycline; KRAB, transcription repressor Krüppel associated box for CRISPRi; VPR, transcription activators VP64-p65-Rta for CRISPRa. F CRISPRi/a MDA-MB-231 xenograft tumor growth in NSG mice (n=9/group). Solid black arrows indicate Dox injections. G xenograft tumors and weights at day 28. I the expression levels of FOXP3 in xenograft tum [file 12943_2021_1472_MOESM1_ESM.docx]

**Dual CRISPR Interference and Activation for Targeted Reactivation of X-linked** **Endogenous *FOXP3* in Human Breast Cancer Cells**

Xuelian Cui, Chao Zhang, Zhifang Xu, Shuaibin Wang, Xin Li, Erica Stringer-Reasor, Sejong Bae, Leiping Zeng, Dehua Zhao, Runhua Liu, Lei S Qi, and Lizhong Wang


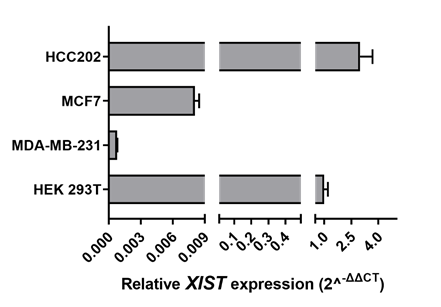


**Figure S1. Expression of *XIST* in human embryonic kidney (HEK) 239T cells and breast cancer cell lines.** The expression levels of *XIST* were assessed by qPCR. The fold change in expression was calculated using the 2^-ΔΔ Ct^ method with *GAPDH* mRNA as an internal control. Data are presented as means ± standard division (SD). HEK 293T, a human embryonic kidney 293 cell line with the SV40 T-antigen; MCF7, a human estrogen receptor (ER)-positive breast cancer cell line; MDA-MB-231, a human triple-negative breast cancer (TNBC) cell line; HCC202, a human epidermal growth factor receptor 2 (HER2)-positive breast cancer cell line. All experiments were repeated three times.


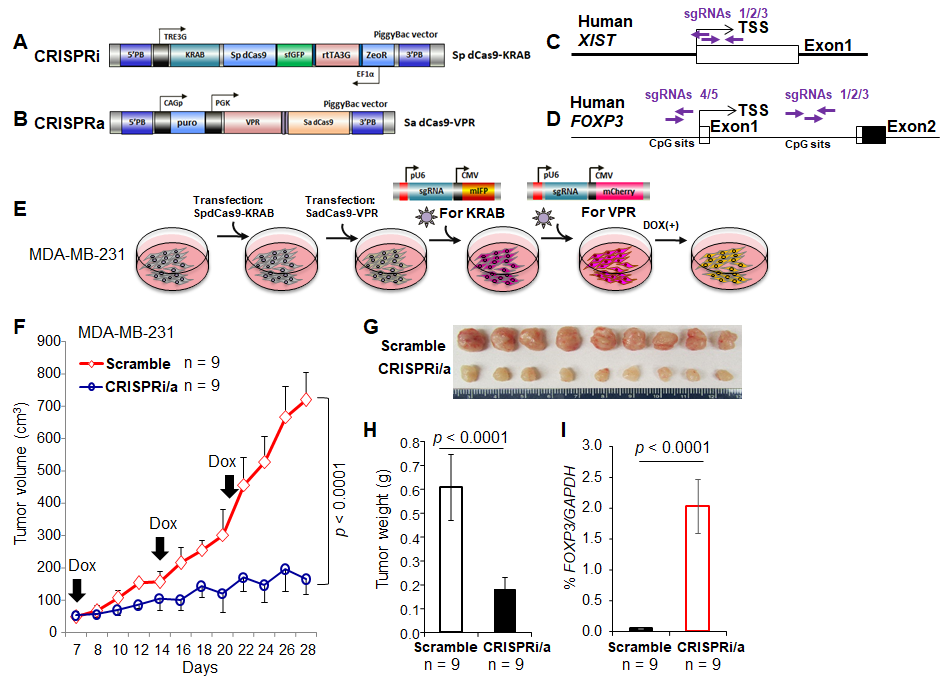


**Figure S2.** **CRISPRi/a DNA construction, experimental procedure,** **and targeted reactivation of endogenous *FOXP3* *in vivo* in activation of endogenous *FOXP3* and repression of *XIST* in human breast cancer MDA-MB-231 cells.** **A, B,** diagrams showing the constructs of CRISPRi/a, including *S. pyogenes* (Sp) dCas9-KRAB (SpdCas9-KRAB) and *S. aureus* (Sa) dCas9-VPR (SadCas9-VPR) used in the experiment. **C,** sgRNAs 1/2/3 targeted to the -50 to +300 bp upstream of the transcription start site of the *XIST* locus for transcription repression. **D,** sgRNAs 1/2/3/4/5 targeted to the two CpG sites of the *FOXP3* proximal promoter and the intron 1 regions for transcription activation. **E,** CRISPRi/a experimental procedure for the co-transduction of *XIST* (mIFP)- and *FOXP3* (mCherry)-sgRNAs, Dox induction, and targeted cell sorting of SpdCas9-KRAB (GFP after Dox) and SadCas9-VPR stably expressing MDA-MB-231 cells. CRISPRi, CRISPR interference; CRISPRa, CRISPR activation; sgRNA, single guide RNA; Dox, doxycycline; KRAB, transcription repressor Krüppel associated box for CRISPRi; VPR, transcription activators VP64-p65-Rta for CRISPRa. **F,** CRISPRi/a MDA-MB-231 xenograft tumor growth in NSG mice (n=9/group). Solid black arrows indicate Dox injections. **G,** xenograft tumors and weights at day 28. **I,** the expression levels of *FOXP3* in xenograft tumors were assessed by qPCR. The fold change in expression was calculated using the 2^-ΔΔ Ct^ method with *GAPDH* mRNA as an internal control. Data are presented as means ± SD. *p* values by a two-way ANOVA or a two-tailed *t*-test.


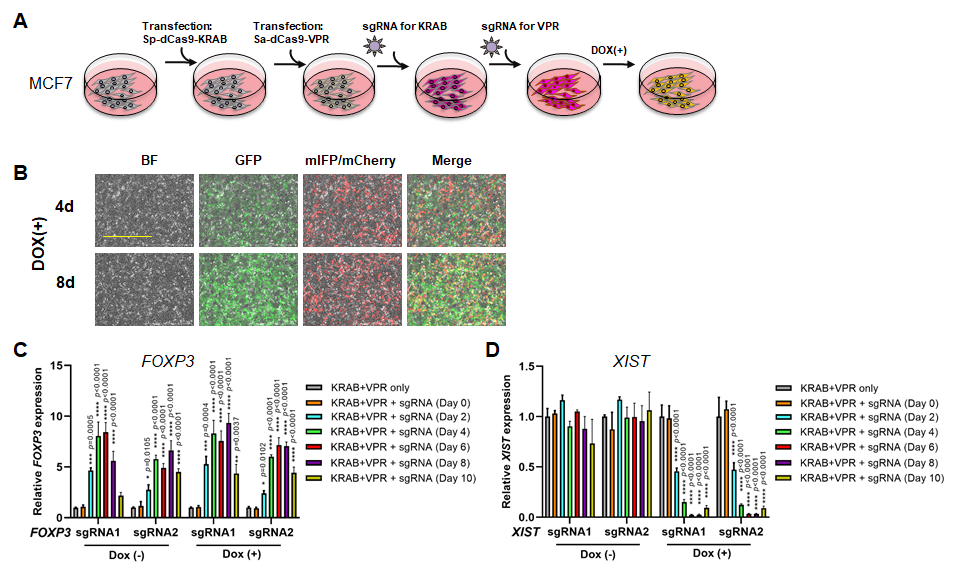


**Figure S3.** **Assessment of CRISPRi/a to activate endogenous *FOXP3* and repress *XIST* in human breast cancer MCF7 cells.** **A,** CRISPRi/a experimental procedure for the co-transduction of *XIST* (mIFP)- and *FOXP3* (mCherry)-sgRNAs and expression of SpdCas9-KRAB (GFP) by Dox induction in CRISPRi/a MCF7 cells. **B,** efficacy of co-transduction of the *XIST-* and *FOXP3-*sgRNAs in CRISPRi/a cells before and after Dox induction at days 4 and 8 as determined by fluorescence microscopy. Scale bar, 1,000 μm. **C, D,** quantitative expression analysis of *FOXP3* and *XIST* before and after sgRNA transduction and Dox induction of CRISPRi/a cells at days 0, 2, 4, 6, 8, and 10 as determined by qPCR. The fold change in expression was calculated using the 2^-ΔΔ Ct^ method with *GAPDH* mRNA as an internal control. Data are presented as the means ± SD. *p* values by ANOVA followed by Tukey's *post hoc* test *vs.* the KRAB+VPR-only group. All experiments were repeated three times.


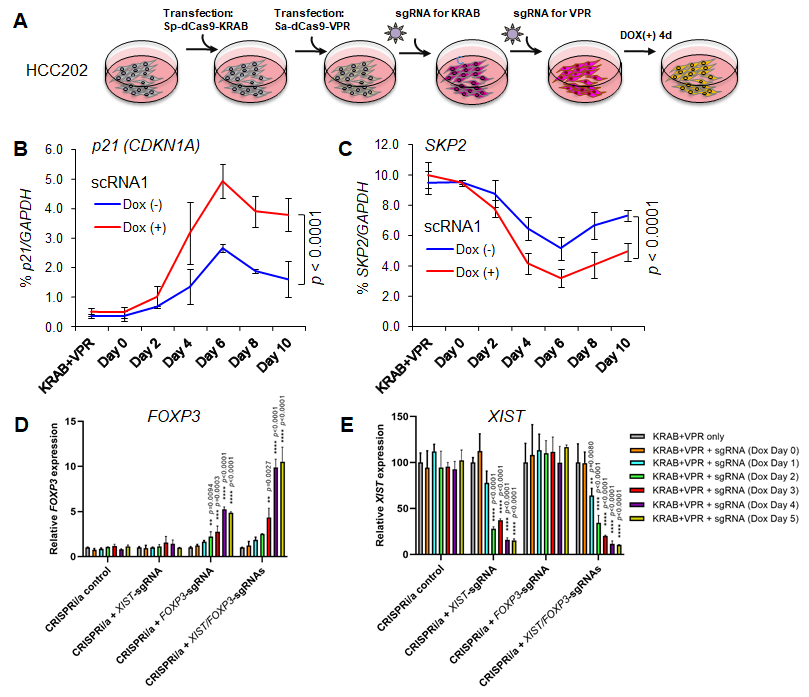


**Figure S4.** **CRISPRi/a DNA construction and experimental procedure** **and** a**ssessment of CRISPRi/a to repress *XIST* in activation of endogenous *FOXP3* and repression of *XIST* in human breast cancer** **HCC202 cells.** **A,** CRISPRi/a experimental procedure for the co-transduction of *XIST* (mIFP)- and *FOXP3* (mCherry)-sgRNAs and Dox induction (GFP for SpdCas9-KRAB) in CRISPRi/a HCC202 cells. **B, C,** quantitative expression analysis of *p21* and *SKP2* by qPCR in CRISPRi/a HCC202 cells with or without Dox at days 0, 2, 4, 6, 8, and 10. **D, E,** quantitative expression analysis of *FOXP3* and *XIST* by qPCR in CRISPRi/a HCC202 cells with or without Dox. The fold change in expression was calculated using the 2^-ΔΔ Ct^ method with *GAPDH* mRNA as an internal control. Error bars, SD. *p* values by a two-way ANOVA or one-way ANOVA followed by Tukey's analysis. All experiments were repeated three times.


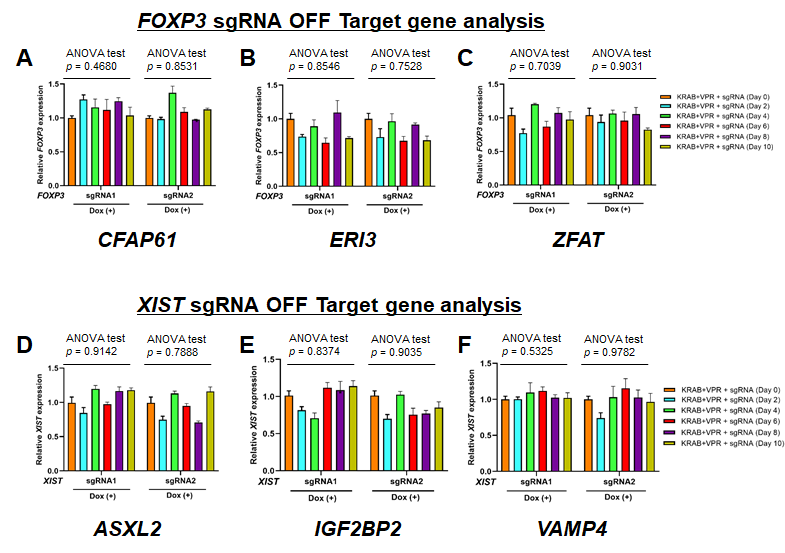


**Figure S5.** **Assessment of** **potential off-target** **genes of *FOXP3* and *XIST* sgRNAs in CRISPRi/a HCC202 cells.** Quantitative expression analysis of the potential off-target genes of *FOXP3* sgRNAs (**A-C**) and *XIST* sgRNAs (**D-F**) before and after sgRNA transduction and Dox induction in CRISPRi/a HCC202 cells at days 0, 2, 4, 6, 8, and 10 as determined by qPCR. The fold change in expression was calculated using the 2^-ΔΔ Ct^ method with *GAPDH* mRNA as an internal control. Data are presented as the means ± SD. * *p* < 0.05 by one-way ANOVA followed by Tukey's *post hoc* test. All experiments were repeated three times.


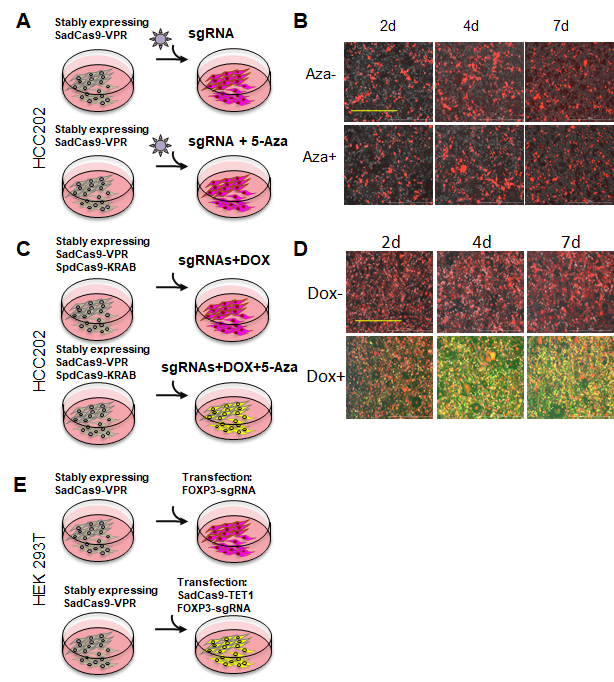


**Figure S6. Experimental procedure and efficacy of transduction in various cell models. A,** the CRISPRi/a experimental procedure for the transduction of *FOXP3* sgRNA (mCherry) with or without 5-Aza-CdR (5-Aza) in CRISPRa HCC202 cells. **B,** efficacy of transduction of *FOXP3* sgRNA in CRISPRa HCC202 cells before and after 5-Aza treatment at days 2, 4, and 7 as determined by fluorescence microscopy. Scale bar, 100 μm. **C,** CRISPRi/a experimental procedure for the co-transduction of *XIST* (mIFP)- and *FOXP3* (mCherry)-sgRNAs with or without 5-Aza and Dox for CRISPRa HCC202 cells. **D,** efficacy of co-transduction of the *FOXP3* sgRNA in CRISPRa HCC202 cells before and after 5-Aza and Dox treatment at days 2, 4, and 7 as determined by fluorescence microscopy. Scale bar, 1,000 μm. **E,** CRISPRa experimental procedure for the transduction of *FOXP3* sgRNA (mCherry) with or without SadCas9-TET1 (GFP) into CRISPRa HEK 293T cells.


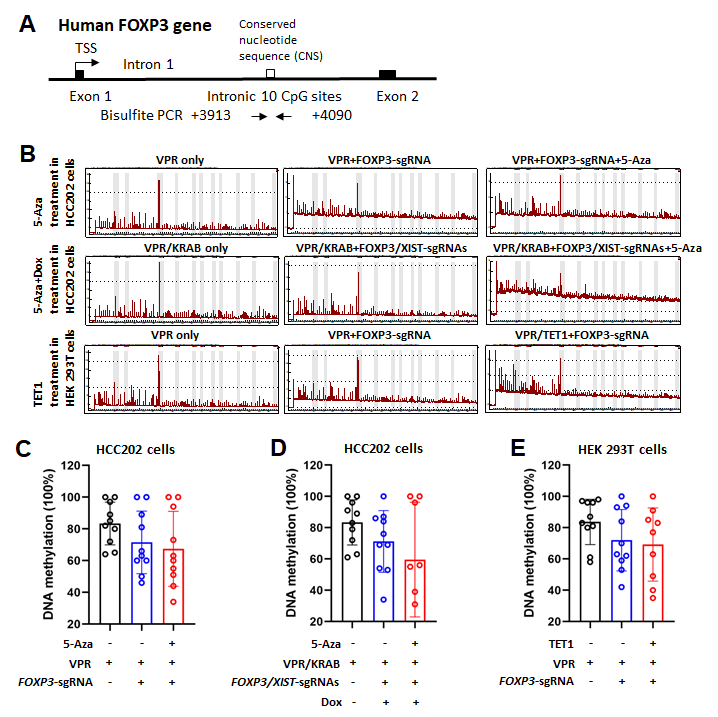


**Figure S7. DNA methylation status** **of the rich CpG sites in** **conserved CNS of *FOXP3* intron 1 during CRISPRi/a**-**mediated activation of *FOXP3*** **in human** **female cells.** **A,** diagram of the 10 CpG sites in conserved CNS of *FOXP3* intron 1 and bisulfite PCR design for DNA methylation pyrosequencing analysis. **B,** DNA methylation analysis by pyrosequencing for CRISPRa HCC202 cells, CRISPRi/a HCC202 cells, and CRISPRa HEK 293T cells with various treatments. Pyrosequencing was performed to measure the methylation levels at 10 CpG sites in the conserved CNS of *FOXP3* intron 1 using the PyroMark Q96 ID pyrosequencer. **C-E,** average levels of DNA methylation in CRISPRa HCC202 cells, CRISPRi/a HCC202 cells, and CRISPRa HEK 293T cells before and after various treatments. Data are presented as the means ± SD. 5-Aza, 5-Aza-2’-deoxycytidine. All experiments were repeated three times.


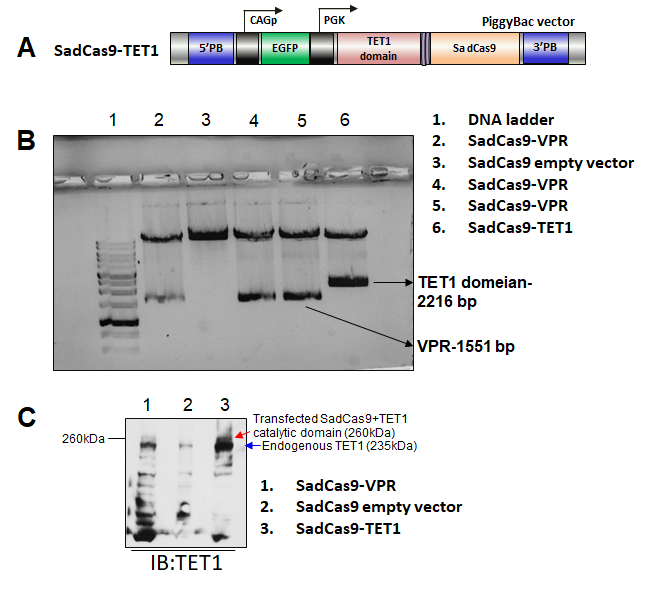


**Figure S8. Establishment of** **SadCas9-TET1 DNA constructs. A,** schematic construction of the SadCas9-TET1 vector used in the experiment. **B,** horizontal gel electrophoresis analysis of bands of the TET1 catalytic domain and VPR digested from SadCas9-TET1 and SadCas9-VPR vectors, respectively. Molecular sizes of the 10-kb DNA ladder are indicated on the left side. **C,** protein expression of TET1 after transfection into HEK 293T cells. The SadCas9-TET1 vector was transiently transfected into HEK 293T cells. The red arrow indicates the size of the SadCas9-TET1 catalytic domain. The blue arrow indicates the full size of the endogenous TET1 protein. IB, Immunoblotting.


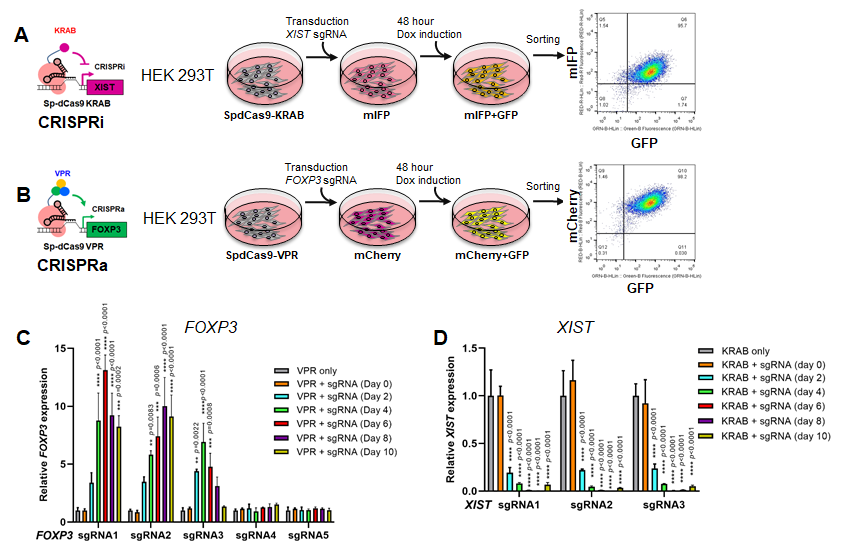


**Figure S9. Targeted activation of *FOXP3* and inactivation of *XIST* in human embryonic kidney (HEK) 293T cells. A,** CRISPRi experimental procedure for the transduction of *XIST* sgRNA (mIFP), Dox induction, and targeted cell sorting of SpdCas9-KRAB (GFP after Dox) stably expressing HEK 293T cells. **B,** CRISPRa experimental procedure for the transduction of *FOXP3* sgRNA (mCherry), Dox induction, and targeted cell soring of SpdCas9-KRAB (GFP after Dox) stably expressing HEK 293T cells. **C, D,** quantitative expression analysis of *XIST* and *FOXP3* by qPCR of CRISPRi and CRISPRa cells, respectively. After Dox (1.0 μg/ml) induction, the expression levels of *XIST* and *FOXP3* in the transduced cells were determined at days 0, 2, 4, 6, 8, and 10. The fold change in expression was calculated using the 2^-ΔΔ Ct^ method with *GAPDH* mRNA as an internal control. Data are presented as the means ± standard deviation (SD). *p* values by a one-way ANOVA test. CRISPRi, CRISPR interference; CRISPRa, CRISPR activation; sgRNA, single guide RNA; Dox, doxycycline; KRAB, transcription repressor Krüppel associated box for CRISPRi; VPR, transcription activators VP64-p65-Rta for CRISPRa. All experiments were repeated three times.


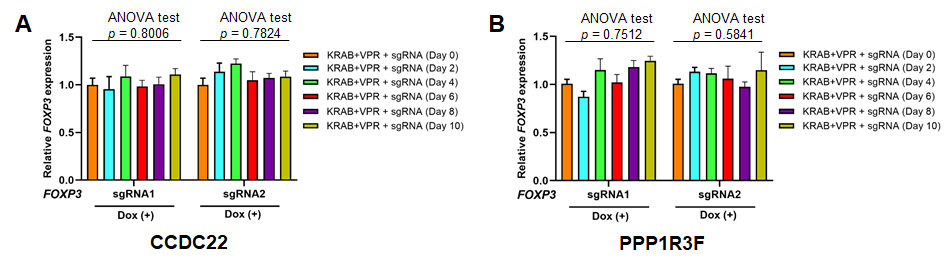


**Figure S10. Effect of CRISPRi/a-mediated activation of *FOXP3* on expression of its** **neighboring genes in HCC202 cells.** *PPP1R3F* and *CCDC22* are two *FOXP3* neighboring genes at Xp11.23. Quantitative expression analysis of *CCDC22* (**A**) and *PPP1R3F* (**B**) before and after sgRNA transduction and Dox induction in the CRISPRi/a cells at days 0, 2, 4, 6, 8, and 10 as determined by qPCR. The fold change in expression was calculated using the 2^-ΔΔ Ct^ method with *GAPDH* mRNA as an internal control. Data are presented as the means ± SD. *p* values by one-way ANOVA followed by Tukey's *post hoc* test. All experiments were repeated three times.


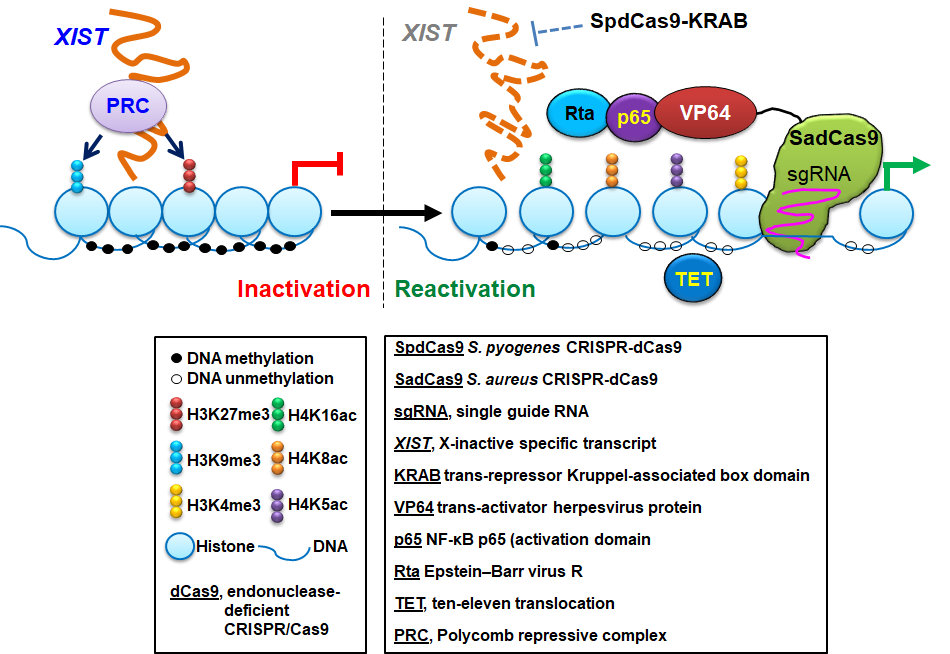


**Figure S11. Targeted reactivation of the X-linked endogenous FOXP3 gene from X chromosome inactivation (XCI) in female cells.** PRC (polycomb repressive complex) 1 or 2 recruits *XIST* RNA and promotes epigenetic modifications that block X-linked FOXP3 gene transcription on the inactive X chromosome. DNA binding by SadCas9-VPR (VP64/p65/Rta) and SadCas9-TET to the *FOXP3* intron 1 enhancer, and subsequent epigenetic modifications, in conjunction with the SpdCas9-KRAB to the *XIST* promoter, reactivates X-linked FOXP3 gene transcription from XCI.
