# Supplementary material for: Dual CRISPR interference and activation for targeted reactivation of X-linked endogenous FOXP3 in human breast cancer cells
Source: Mol Cancer. 2022 Feb 7;21:38. doi: 10.1186/s12943-021-01472-x (PMC8819949; doi:10.1186/s12943-021-01472-x)
Supplement: Supplementary file 2 — Additional file 2. Materials and Methods [file 12943_2021_1472_MOESM2_ESM.docx]

**Dual** **CRISPR Interference and Activation for Targeted Reactivation of X-linked** **Endogenous *FOXP3* in Human Breast Cancer Cells**

Xuelian Cui, Chao Zhang, Zhifang Xu, Shuaibin Wang, Xin Li, Erica Stringer-Reasor, Sejong Bae, Leiping Zeng, Dehua Zhao, Runhua Liu, Lei S. Qi, and Lizhong Wang

**Materials and Methods**

**DNA construction for CRISPR Interference and Activation (CRISPRi/a)**

Individual CRISPRi/a PiggyBac DNA constructs for endonuclease-deficient CRISPR/Cas9 (dCas9) effectors used in this study are described in Table S4. To assemble the doxycycline (Dox)-inducible dCas9-effector constructs, human codon-optimized *S. pyogenes* dCas9 was fused at the C-terminus with an HA tag and two SV40 nuclear localization signals, followed by the effector [1]. The VP64-p65-Rta (VPR) effector [2] was assembled by fusing the activation domain of VP64 with the activation domain of p65 and Epstein-Barr virus R (Rta) with two glycine-serine linkers. An extra SV40 nuclear localization signal was inserted between VP64 and p65. To pick out cell clones containing this vector, the puromycin-resistance gene was added to the N-terminal of the VPR effector followed by a CAG promoter. The *S. Aureus* dCas9 (gift from Feng Zhang, Addgene plasmid no. 61594) constructs and the VPR effector were driven by a PGK promoter. The catalytic domain of TET1 was amplified by PCR without any mutation and cloned onto the vector instead of the VPR effector. For visualization, enhanced fluorescent green protein (GFP) was fused to the N-terminal of the effector to replace the puromycin-resistance gene.

The *X-inactive specific transcript* (*XIST*)-single guide RNAs (sgRNAs) and *FOXP3*-sgRNAs were designed to target the -50 to +300 bp upstream of the transcription start site of the *XIST* locus for Krüppel-associated box (KRAB) transcription repression and target to the two CpG-rich sites of the promoter and intron 1 of the *FOXP3* locus for VPR transcription activation or TET1 DNA demethylation. As shown in Table S4, for targeted repression of *XIST,* we constructed three *XIST*-sgRNAs to the pHR lentiviral U6-based expression vector (pSLQ2837), which expresses monomeric infrared fluorescent protein (​mIFP). Likewise, we constructed five *FOXP3*-sgRNAs to the pHR lentiviral U6-based expression vector (pSLQ2806), which expressed monomeric red fluorescent protein (mCherry). Alternative sgRNA sequences (Table S5) were generated by PCR reactions with Q5-High-Fidelity DNA-polymerase (NEB) according to the manufacturer's protocol. We used pSLQ2837 (for *XIST* sgRNA)/pSLQ2806 (for *FOXP3* sgRNA) as PCR templates to the sgRNA using the forward primer and the universal reverse primer. Then, the purified PCR product was introduced by InFusion cloning into the pSLQ2837/pSLQ2806 backbone vector, which was digested with *BstX*I and *Xho*I as previously described [1]. All DNA constructs of sgRNAs were confirmed by DNA Sanger sequencing.

**Cell culture and transfection**

HEK 293T, MCF7, and MDA-MB-231 cell lines (ATCC) were cultured in high-glucose DMEM media supplemented with 10% fetal bovine serum (FBS, Thermo Fisher Scientific, Waltham, MA). The HCC202 (also known as CRL-2316, ATCC) cell line was routinely grown in RPMI-1640 medium supplemented with 10% FBS. All cell lines were cultured at 37 °C with 5% CO_2_ for less than six months, were authenticated by examination of morphology and growth characteristics, and were confirmed to be mycoplasma-free. Short tandem-repeat analysis for DNA fingerprinting was also used to verify the cell lines. To achieve high transfection efficiencies, HEK 293T, MCF7, MDA-MB-231, and HCC202 cell lines were stably generated with the Piggybac-based dCas9-effector constructs and then followed by transduction with suitable sgRNA vectors. One day before transfection, the cultured cells were seeded in 6-well plates and were grown to about 80% confluency on the day of transfection. To integrate the Piggybac-based dCas9-effector constructs to the genome, the PiggyBac plasmid containing the dCas9-effector and Super PiggyBac transposase (Systems Biosciences, Palo Alto, CA) plasmid were co-transfected at a ratio of 2.5:1 using Lipofectamine 3000 (Thermo Fisher Scientific). After incubation with the transfection complexes for 48 hours, stably integrated cells were selected by Zeocin and GFP for CRISPRi and puromycin for the CRISPRa vector for 14 days. Then, the transfected cells were isolated as single clones and confirmed by Western blots.

To evaluate the effectiveness of the sgRNAs-induced dcas9 system, the stably integrated cells containing the CRISPRi or CRISPRa vector were subjected to transient transduction of sgRNA vectors. Cells were harvested for RNA isolation at 2, 4, 6, 8, and 10 days post-transduction and analyzed by reverse transcriptase qPCR. To establish the cells containing CRISPRi/a vectors and sgRNA vectors, the most effective sgRNA vectors for CRISPRi/a were lentivirally transduced into the stably integrated cells containing the CRISPRi/a vector. For lentivirus generation, HEK 293T cells were seeded into two 10-cm plates and were grown to about 80% confluency on the day of transduction. Then, individual lentiviral sgRNA vectors were co-transduced with two lentiviral packaging plasmids, psPAX2 and pMD2G (Addgene, Cambridge, MA) at a ratio of 2:2:1 using polyethylenimine (Polysciences, Warrington, PA). After 18 hours of transduction, the medium was exchanged with fresh medium, and, at 48 hours after replacing the medium, the packaged lentivirus supernatant was collected and passed through a 0.45-µm filter. Targeted cells were transduced with packaged sgRNA lentiviruses and were sorted, at 48 hours post-transduction, with mIFP or mCherry by use of a BD FACS Aria II flow cytometer (Thermo Fisher Scientific). Then, the positive mIFP or mCherry fluorescence cells containing CRISPRi/a vectors were cultured for four passages for use in subsequent experiments.

**Establishment of** **CRISPRi/a-based platform for targeted** **repression of *XIST* and activation of *FOXP3***

dCas9-mediated gene-specific epigenetic modifications are based on dCas9 fusion to transcriptional and epigenetic effectors [3]. dCas9 fusion to the transcriptional repressor KRAB domain [4] is used for CRISPRi-mediated repression of gene expression. In contrast, dCas9 fusion to tripartite transcriptional activators VPR [5, 6] is used for CRISPRa-mediated activation of gene expression. To induce the transcription of X-linked endogenous *FOXP3* in female cells, we developed a dCas9-based platform for simultaneous non-coding RNA *XIST* repression and *FOXP3* activation within the same cells using our previously established method [7]. Briefly, DNA constructs consist of a coherent set of pSLQ1922 for *Streptococcus pyogenes* (Sp) dCas9 (SpdCas9)-KRAB [doxycycline (Dox) inducible], pSLQ1932 for SpdCas9-VPR (Dox inducible), pSLQ2840 for *Staphylococcus aureus* (Sa) dCas9 (SadCas9)-VPR, modified pSLQ2840 for SadCas9-TET1, pSLQ2837 for *XIST* sgRNA, and pSLQ2806 for *FOXP3* sgRNA (Figs. S2A, S2B and S8A and Table S4). The three *XIST* sgRNA1/2/3 were established in our previous study [8] (Fig. S2C and Table S5). For targeted activation of *FOXP3,* we designed five sgRNAs (Table S5) at the two CpG-rich loci in the proximal promoter region of the transcription start site and the conserved non-coding sequence (CNS) region of intron 1 [9-12] of *FOXP3* (Fig. S2D). The *XIST-* and *FOXP3-*sgRNAs guide the SpdCas9-KRAB and SadCas9-VPR fusions, respectively, to the target sequences at the *XIST* (proximal promoter region) and *FOXP3* (two CpG sites) loci, respectively [7, 8, 12-19].

Two human embryonic kidney HEK 293T stable cell lines derived from a female fetus that stably expressed SpdCas9-KRAB or SpdCas9-VPR have been established in our previous study [20] (Table S2). First, we validated the repression of *XIST* in our CRISPRi system using the established SpdCas9-KRAB-stably expressing HEK 293T cell line. Three sgRNAs were designed to target the promoter and enhancer loci of *XIST* (Fig. S2C). With CRISPRi HEK 293T cells, we validated the repression of *XIST* transcription by the *XIST*-sgRNAs 1/2/3 (Fig. S9D) [8]. Next, we validated the activation of *FOXP3* transcription in our CRISPRa system using the SpdCas9-VPR-stably expressing HEK 293T cell line. Five sgRNAs were designed to target the promoter and enhancer loci of *FOXP3* (Fig. S2D). After addition of Dox, levels of the *FOXP3* transcript were increased by 3- to 13-fold by use of the *FOXP3*-targeting sgRNAs 1/2/3 in the CpG-rich site of the *FOXP3* intron 1, but not in the proximal promoter region of the transcription start site (Figs. S9C). This result validated the efficacy of our CRISPRi/a-based platform for targeted repression of *XIST* and activation of *FOXP3* in human HEK 293T cells.

**Quantitative real-time PCR (qPCR)**

Total RNA was extracted from cells using the TRIzol Reagent (Thermo Fisher Scientific), and 1 µg of total RNA was reverse-transcribed with High-Capacity cDNA Reverse Transcription kits (Thermo Fisher Scientific). For each qPCR reaction, primers and 20 ng of cDNA were mixed in a 10-µl template and amplified using an SYBR Green Supermix kit (Thermo Fisher Scientific) with a Light Cycler 480 II instrument (Roche, Basel, Switzerland). The primer sequences are listed in Table S5. The fold-increases of mRNA expression of the genes of interest were calculated using the 2^-ΔΔ Ct^ method with *GAPDH* mRNA as an internal control.

**Co-immunoprecipitation (Co-IP) and Western blots**

At about 90% confluency, cells on 10-cm plates were washed with cold PBS and lysed in ice-cold buffer (20 mM Tris–HCl, pH 8.0), 150 mM NaCl, 1 mM EDTA, 1% NP-40) supplemented with protease inhibitors (Sigma-Aldrich, St. Louis, MO) on ice for 10 min. After centrifuging of the preparations, the lysates were aliquoted into two tubes and incubated with either the designated antibody or an appropriate IgG control for 16 hours at 4°C. Then, the immune complexes were precipitated with Rec-protein G-Sepharose^TM^ (Thermo Fisher Scientific), and the proteins were eluted from the pelleted beads with 2x SDS sample buffer for subsequent Western blot analyses. For Western blotting, 30 µg of whole-cell lysates was separated on SDS-polyacrylamide gels and transferred to polyvinylidene difluoride membranes (Millipore, Burlington, MA). The membranes were incubated with appropriate primary antibodies, followed by either an anti-rabbit or anti-mouse IgG HRP-linked secondary antibody. The details of antibodies are listed in Table S6. Then, after incubation of the membranes with chemiluminescence reagents, the HRP signal was evaluated by exposure of the membranes to X-ray films.

**Fluorescence-activated cell sorting (FACS)**

For obtaining stably integrated cells with the CRISPRi vector, cells were gated for GFP-positive expression, and the population of interest was sorted and cultured in medium containing Zeocin (1 µg/ml). For sorting of cell pools with sgRNA vectors, cells were gated for mIFP- or mCherry-positive expression for the CRISPRi or CRISPRa system, and the population of the highest positive reporter fluorescent protein was collected and cultured for four passages for subsequent experiments. Each positive fluorescence population was gated, normalized to the un-transduced control cells by an BD FACS Aria II flow cytometer, and analyzed by FlowJo software (BD Biosciences, Ashland, OR).

**RNA fluorescence in situ hybridization (FISH)**

RNA-FISH was performed as previously described [21, 22]. Cells were seeded on glass coverslips in 3.5-cm plates for 2 days. After washing with Hank’s balanced salt solution (Thermo Fisher Scientific), coverslips were rinsed with Cytoskeletal buffer (CSK buffer) on ice. For extraction, coverslips were kept in CSK buffer with 0.5% Triton X-100 and 2 mM VRC (vanadyl ribonucleoside complex, Sigma-Aldrich) for 10 min on ice and were treated with 4% paraformaldehyde solution for 8 min of fixation. To detect human *XIST*, an appropriate DNA probe G1A (Addgene, plasmid no. 24690), encompassing approximately 10 kb of genomic DNA of the XIST gene, was processed with Digoxigenin-11-dUTP (Roche) and Nick-translation mix (Roche) [23]. For hybridization, 50 ng of labeled probe was mixed with 10 μg of human Cot-1 DNA (Roche) and 10 μg each of salmon sperm DNA and *E. Coli* tRNA. The probe was air-dried and denatured on an 80°C heat block with 100% formamide. Then, coverslips were hybridized on the slides with 20 μl of hybridization buffer containing 80 U/μl of RNasin overnight. On the following day, the coverslips were rinsed in 2× saline-sodium citrate (SSC with 50% formamide) at 37°C, in 2×SSC at 37°C, in 1×SSC at room temperature on a shaker, and finally in 4×SSC at room temperature for 2 min to equilibrate cells before detection. Then, coverslips were incubated with a secondary antibody of anti-digoxigenin fluorescein (Roche) for 1 hour at 37°C and counterstained with 4’,6-diamidino-2-phenylindole (DAPI) for 1 min. Finally, coverslips were mounted using Vectashield (Vector Labs, Burlingame, CA) mounting media and imaged with a BX43 microscope (Olympus, Tokyo, Japan).

**Immunofluorescence imaging**

The stably integrated cells containing CRISPRi or CRISPRa vector or both CRISPRi and CRISPRa vectors were seeded on 6-well plates for transduction with sgRNA vectors. At selected times, the positive reporter fluorescent signal of cells was imaged with a Lionheart FX Automated Microscope (BioTek, Winooski, VT). Selected positions of each image were acquired under a bright field, with GFP and Texas Red filter cubes, and merged for visualization of cells containing both fluorescent signals.

**DNA methylation analysis**

DNA methylation status was determined by PCR analysis of bisulfite-modified genomic DNA using pyrosequencing (PyroMark Q96 ID; Qiagen, Germantown, MD) according to the manufacturer’s instructions. The primers were designed by use of the Pyromark Assay Desing Software package (Qiagen), and 10 CpG sites of the *FOXP3* intron region were selected for quantitative DNA methylation analysis. These CpG sites were associated with activation of *FOXP3* by CRISPRi/a. DNA bisulfite conversion was performed by use of EpiTect Bisulfite Kits (Qiagen) followed by PCR amplification with primers. The pre-PCR products were used for bisulfite pyrosequencing to determine DNA methylation levels for each CpG site. Also, the means of DNA methylation levels in the 10 CpG sites were calculated to obtain an average percentage of methylation. The primers used are described in the Table S5.

**Chromatin immunoprecipitation (ChIP) assays**

ChIP assays were performed as previously described [24]. Stable cells with CRISPRi and CRISPRa vectors were transduced with 12 µg of sgRNA vectors for CRISPRi and CRISPRa effector on 10-cm plates using Lipofectamine 3000. After 96 hours of transduction, the cross-linked samples were sonicated in SDS Lysis Buffer (1% SDS, 10 mM EDTA, and 50 mM Tris-HCl, pH 8.1) using a Fisherbrand^TM^ Model 120 Sonic Dismembrator (Thermo Fisher Scientific) to 200-1,000bp fragments. Sheared samples were centrifuged for 10 min at 13,000 rpm and 4°C, and the pellets were diluted with ChIP dilution buffer (0.01% SDS; 1.1% Triton X-100; 1.2 mM EDTA; 16.7 mM Tris-HCl, pH 8.1; and 167 mM NaCl). ChIP enrichment of each sample was conducted by adding 2-4 μg of each antibody. Then, the immune complexes were pulled down with Rec-protein G-Sepharose^TM^ (Thermo Fisher Scientific), and the DNA was eluted from the pelleted protein G agarose/antibody/protein complex with elution buffer (1% SDS, 0.1 M NaHCO_3_). ChIP-qPCR was performed to quantify the amounts of immune-enriched DNA fragments. The primers and antibodies are shown in Tables S5 and S6.

**CRISPR off-target analysis**

To exclude the potential off-target effects of each sgRNA, we screened the sequences of *XIST* and *FOXP3* sgRNAs using the off-target searching tool (Cas-OFFinder, Daejeon, South Korea, <http://www.rgenome.net/cas-offinder>). Unbiased off-target screening was performed on the human reference genome GRCh38 for canonical SpCas9 PAM sites using 20-base-pair target sites without protospacer adjacent motif (PAM) sequences [25]. The criteria for settings were confined to four or fewer mismatches with no DNA or RNA bulge. The total number and the possible binding loci of off-target genes of *XIST* and *FOXP3* sgRNAs are summarized in Tables S3a-d. Subsequently, to assess the specificity of sgRNA-induced effects on target sites, qPCR was conducted to measure the mRNA expression of off-target genes (mismatches ≤ 3) of *XIST* and *FOXP3* sgRNAs and the nearest neighboring genes (*PPP1R3F* and *CCDC22*) to the *FOXP3* locus. Primers for qPCR and sgRNAs are shown in Table S5.

**Xenogeneic transplantation**

NSG mice were purchased from the Jackson Laboratory (Bar Harbor, ME). All animal experiments were conducted following the protocol approved by the Institutional Animal Care and Use Committee of University of Alabama at Birmingham (UAB; Birmingham, AL). For an orthotopic breast cancer model, 1 × 10^6^ breast cancer cells were injected into the fourth mammary fat pads of 8-week-old female NSG mice. Xenograft tumor growth was assessed for 4 weeks after tumor cell injection. Mice were intraperitoneally injected with Dox (2.5 mg/kg weekly) to induce CRISPR-dCas9 expression. Tumor growth was measured with calipers every other day, and their volumes were determined as (width^2^ × length)/2 [26, 27].

For an intratibial breast cancer model, NSG mice were anesthetized with isoflurane, then moved to nose cones and maintained under anesthesia. Hind legs of mice were cleaned with 10% povidone/iodine swab/solution, followed by ethanol, repeating two times. If fur was present, the legs were further depilated before cleaning. The lateral malleolus, medial malleolus, and lower half of the tibia were gently grasped with forefinger and thumb, and the leg was bent. With the ankle and leg of mice firmly grasped, a 28-g ½ needle was inserted under the patella, through the middle of the patellar ligament, and into the anterior intercondylar area in the top of tibia. When the needle was inserted into the tibia, it was guided through the growth plate by steady, firm pressure with slight drilling action. Upon penetration of the tibial growth plate, the needle encountered markedly less resistance. Then, the needle was moved using a gentle, lateral movement to ensure that it was in the tibia and through the growth plate. Meanwhile, the plunger was slowly depressed, and 10 μl of cell solution (1×10^5^) was injected. At this point, little to no resistance was felt. At last, the needle was slowly extracted. The mouse was removed from anesthesia and kept on a heating pad until recovery. Xenograft tumor growth was monitored by imaging using an IVIS Lumina Series III (PerkinElmer, Akron, OH). The numbers of surface tumor lesions over all lobes of the lungs were scored as metastatic nodules.

**Statistical analyses**

Differences in outcomes between two groups were compared by two-sided *t*-tests. Analysis of variance (ANOVA), one- and two-way, were used to test for overall differences, followed by a Dunnett *post hoc* test for differences between groups. All data were entered into an Access database using Excel (Microsoft 365 ProPlus) and analyzed with SPSS (version 25; IBM, Armonk, NY) and GraphPad (Prism 8, San Diego, CA).

**References**

1. Gilbert LA, Larson MH, Morsut L, Liu Z, Brar GA, Torres SE, Stern-Ginossar N, Brandman O, Whitehead EH, Doudna JA, et al: **CRISPR-mediated modular RNA-guided regulation of transcription in eukaryotes.** *Cell* 2013, **154:**442-451.

2. Chavez A, Scheiman J, Vora S, Pruitt BW, Tuttle M, E PRI, Lin S, Kiani S, Guzman CD, Wiegand DJ, et al: **Highly efficient Cas9-mediated transcriptional programming.** *Nat Methods* 2015, **12:**326-328.

3. Perez-Pinera P, Kocak DD, Vockley CM, Adler AF, Kabadi AM, Polstein LR, Thakore PI, Glass KA, Ousterout DG, Leong KW, et al: **RNA-guided gene activation by CRISPR-Cas9-based transcription factors.** *Nat Methods* 2013, **10:**973-976.

4. Thakore PI, D'Ippolito AM, Song L, Safi A, Shivakumar NK, Kabadi AM, Reddy TE, Crawford GE, Gersbach CA: **Highly specific epigenome editing by CRISPR-Cas9 repressors for silencing of distal regulatory elements.** *Nat Methods* 2015, **12:**1143-1149.

5. Hilton IB, D'Ippolito AM, Vockley CM, Thakore PI, Crawford GE, Reddy TE, Gersbach CA: **Epigenome editing by a CRISPR-Cas9-based acetyltransferase activates genes from promoters and enhancers.** *Nat Biotechnol* 2015, **33:**510-517.

6. Dominguez AA, Lim WA, Qi LS: **Beyond editing: repurposing CRISPR-Cas9 for precision genome regulation and interrogation.** *Nat Rev Mol Cell Biol* 2016, **17:**5-15.

7. Gao Y, Xiong X, Wong S, Charles EJ, Lim WA, Qi LS: **Complex transcriptional modulation with orthogonal and inducible dCas9 regulators.** *Nat Methods* 2016, **13:**1043-1049.

8. Gilbert LA, Horlbeck MA, Adamson B, Villalta JE, Chen Y, Whitehead EH, Guimaraes C, Panning B, Ploegh HL, Bassik MC, et al: **Genome-Scale CRISPR-Mediated Control of Gene Repression and Activation.** *Cell* 2014, **159:**647-661.

9. Kim HP, Leonard WJ: **CREB/ATF-dependent T cell receptor-induced FoxP3 gene expression: a role for DNA methylation.** *J Exp Med* 2007, **204:**1543-1551.

10. Polansky JK, Kretschmer K, Freyer J, Floess S, Garbe A, Baron U, Olek S, Hamann A, von Boehmer H, Huehn J: **DNA methylation controls Foxp3 gene expression.** *Eur J Immunol* 2008, **38:**1654-1663.

11. Zheng Y, Josefowicz S, Chaudhry A, Peng XP, Forbush K, Rudensky AY: **Role of conserved non-coding DNA elements in the Foxp3 gene in regulatory T-cell fate.** *Nature* 2010, **463:**808-812.

12. Ohkura N, Hamaguchi M, Morikawa H, Sugimura K, Tanaka A, Ito Y, Osaki M, Tanaka Y, Yamashita R, Nakano N, et al: **T cell receptor stimulation-induced epigenetic changes and Foxp3 expression are independent and complementary events required for Treg cell development.** *Immunity* 2012, **37:**785-799.

13. Floess S, Freyer J, Siewert C, Baron U, Olek S, Polansky J, Schlawe K, Chang HD, Bopp T, Schmitt E, et al: **Epigenetic control of the foxp3 locus in regulatory T cells.** *PLoS Biol* 2007, **5:**e38.

14. Baron U, Floess S, Wieczorek G, Baumann K, Grutzkau A, Dong J, Thiel A, Boeld TJ, Hoffmann P, Edinger M, et al: **DNA demethylation in the human FOXP3 locus discriminates regulatory T cells from activated FOXP3(+) conventional T cells.** *Eur J Immunol* 2007, **37:**2378-2389.

15. Schaub B, Liu J, Hoppler S, Schleich I, Huehn J, Olek S, Wieczorek G, Illi S, von Mutius E: **Maternal farm exposure modulates neonatal immune mechanisms through regulatory T cells.** *J Allergy Clin Immunol* 2009, **123:**774-782 e775.

16. Lal G, Bromberg JS: **Epigenetic mechanisms of regulation of Foxp3 expression.** *Blood* 2009, **114:**3727-3735.

17. Lal G, Zhang N, van der Touw W, Ding Y, Ju W, Bottinger EP, Reid SP, Levy DE, Bromberg JS: **Epigenetic regulation of Foxp3 expression in regulatory T cells by DNA methylation.** *J Immunol* 2009, **182:**259-273.

18. Cong L, Ran FA, Cox D, Lin S, Barretto R, Habib N, Hsu PD, Wu X, Jiang W, Marraffini LA, Zhang F: **Multiplex genome engineering using CRISPR/Cas systems.** *Science* 2013, **339:**819-823.

19. Morikawa H, Ohkura N, Vandenbon A, Itoh M, Nagao-Sato S, Kawaji H, Lassmann T, Carninci P, Hayashizaki Y, Forrest AR, et al: **Differential roles of epigenetic changes and Foxp3 expression in regulatory T cell-specific transcriptional regulation.** *Proc Natl Acad Sci U S A* 2014, **111:**5289-5294.

20. Du D, Roguev A, Gordon DE, Chen M, Chen SH, Shales M, Shen JP, Ideker T, Mali P, Qi LS, Krogan NJ: **Genetic interaction mapping in mammalian cells using CRISPR interference.** *Nat Methods* 2017, **14:**577-580.

21. Byron M, Hall LL, Lawrence JB: **A multifaceted FISH approach to study endogenous RNAs and DNAs in native nuclear and cell structures.** *Curr Protoc Hum Genet* 2013, **Chapter 4:**Unit 4 15.

22. Sirchia SM, Tabano S, Monti L, Recalcati MP, Gariboldi M, Grati FR, Porta G, Finelli P, Radice P, Miozzo M: **Misbehaviour of XIST RNA in breast cancer cells.** *PLoS One* 2009, **4:**e5559.

23. Chow JC, Hall LL, Baldry SE, Thorogood NP, Lawrence JB, Brown CJ: **Inducible XIST-dependent X-chromosome inactivation in human somatic cells is reversible.** *Proc Natl Acad Sci U S A* 2007, **104:**10104-10109.

24. Im H, Grass JA, Johnson KD, Boyer ME, Wu J, Bresnick EH: **Measurement of protein-DNA interactions in vivo by chromatin immunoprecipitation.** *Methods Mol Biol* 2004, **284:**129-146.

25. Bae S, Park J, Kim JS: **Cas-OFFinder: a fast and versatile algorithm that searches for potential off-target sites of Cas9 RNA-guided endonucleases.** *Bioinformatics* 2014, **30:**1473-1475.

26. Liu R, Yi B, Wei S, Yang WH, Hart KM, Chauhan P, Zhang W, Mao X, Liu X, Liu CG, Wang L: **FOXP3-miR-146-NF-kappaB Axis and Therapy for Precancerous Lesions in Prostate.** *Cancer Res* 2015, **75:**1714-1724.

27. Wang L, Liu R, Li W, Chen C, Katoh H, Chen GY, McNally B, Lin L, Zhou P, Zuo T, et al: **Somatic single hits inactivate the X-linked tumor suppressor FOXP3 in the prostate.** *Cancer Cell* 2009, **16:**336-346.
